# Significant anthropogenic-induced changes of climate classes since 1950

**DOI:** 10.1038/srep13487

**Published:** 2015-08-28

**Authors:** Duo Chan, Qigang Wu

**Affiliations:** 1 School of Atmospheric Sciences, Nanjing University, 163 Xianlin Ave., Nanjing, Jiangsu, China, 210023

## Abstract

Anthropogenic forcings have contributed to global and regional warming in the last few decades and likely affected terrestrial precipitation. Here we examine changes in major Köppen climate classes from gridded observed data and their uncertainties due to internal climate variability using control simulations from Coupled Model Intercomparison Project 5 (CMIP5). About 5.7% of the global total land area has shifted toward warmer and drier climate types from 1950–2010, and significant changes include expansion of arid and high-latitude continental climate zones, shrinkage in polar and midlatitude continental climates, poleward shifts in temperate, continental and polar climates, and increasing average elevation of tropical and polar climates. Using CMIP5 multi-model averaged historical simulations forced by observed anthropogenic and natural, or natural only, forcing components, we find that these changes of climate types since 1950 cannot be explained as natural variations but are driven by anthropogenic factors.

## Introduction

Anthropogenic forcings, dominated by increasing greenhouse gas (GHG) concentrations, have very likely contributed to global and regional warming since 1950^1^,^2^,^3^,^4^,^5^ and likely affected land precipitation^5^,^6^,^7^,^8^,^9^. Long-term changes in climate classes are also important indicators for climatic changes. The Köppen climate classes are designed to explain observed biome distributions, which have many sharp boundaries due to plant sensitivity to threshold values of average monthly temperature and precipitation and their annual cycle^10^,^11^. Köppen or similar classifications have been used to estimate the potential impacts of past and projected future climate on prevalent ecoregions on regional and global scales^10^,^11^,^12^,^13^,^14^,^15^,^16^,^17^,^18^,^19^. For example, major Köppen climate types are projected to shift strongly toward warmer and drier climates (temperate, tropical and arid), with climate types in 31.4% and 46.3% of the global land area projected to change by 2100 under RCP4.5 and RCP8.5 scenarios, respectively^20^.

However, it is still not clear whether significant changes of climate types are already detectable in observations, and whether such changes can be attributed to external anthropogenic forcing. This study uses updated thresholds^11^ for five major climate classes (Methods) based on climate data at a location, with tests performed in the following order to always assign a unique climate class: Arid (class B), Tropical (class A), Polar (class E), Temperate (class C), Continental (class D). To reduce the probabilistic influence of shorter-term climate variability and elongate the period with available data, a 15-yr running smooth is applied to all variables in both observation and model data. We compute Köppen climate classifications from gridded observation and model data compared to 1950 to develop four indices describing the distribution of climate types: (1) percentage of world land area with a major climate type change from 1950; (2) total area occupied by each major climate type; (3) averaged absolute latitude of each major climate type; and (4) average elevation of each major climate type. Changes of these indices and their statistical significance are first evaluated, and the relative roles of external anthropogenic and natural forcing in these changes are assessed.

We primarily use the University of Delaware (UD) global land 0.5° gridded monthly temperature and precipitation dataset^21^,^22^, updated through 2010, to calculate the Köppen climate type in each grid box. The UD dataset has been used in other studies of Köppen climate classification^20^. Temperature and precipitation fields in the UD dataset are heavily interpolated and the regional averages reflect to a certain degree the changes in data coverage over both time and space. For comparison, we use two other datasets to examine the robustness and consistency of detected changes of climate type indices in the UD dataset, including the University of East Anglia Climate Research Unit (CRU) 0.5° gridded monthly temperature and precipitation dataset (CRU_TS_3.22)^23^ and the Goddard Institute for Space Studies (GISS) 2° × 2° gridded monthly temperature anomaly field^24^. The CRU dataset fills in all grid boxes, but effectively has zero anomalies in grid boxes with no stations within 1200 km, while the GISS temperature dataset counts such grid boxes as “missing.” Such “interpolated” boxes in UD and CRU are almost identical to the “missing” boxes in GISS (see Supplementary Fig. S4). Even with differing procedures to develop gridded values, the annual and long-term values from these datasets agree very well. Because GISS does not provide a precipitation dataset, here the UD precipitation is also averaged to the GISS grid, and “GISS dataset” refers to the GISS temperature and regridded UD precipitation data at 2° resolution.

Model runs are selected from the CMIP5 dataset^25^, including preindustrial control (PI-CTL) runs to estimate natural variability statistics, historical runs to distinguish natural and anthropogenic factors, and projection runs to estimate future climate type changes (Supplementary Table S1). For each major climate type, we apply a one-sided local significance test to identify whether the observed trends of the four indices are significantly different from zero at the 5% significance level.

### Observed changes of climate type indices from the UD dataset

**Figure 1:**
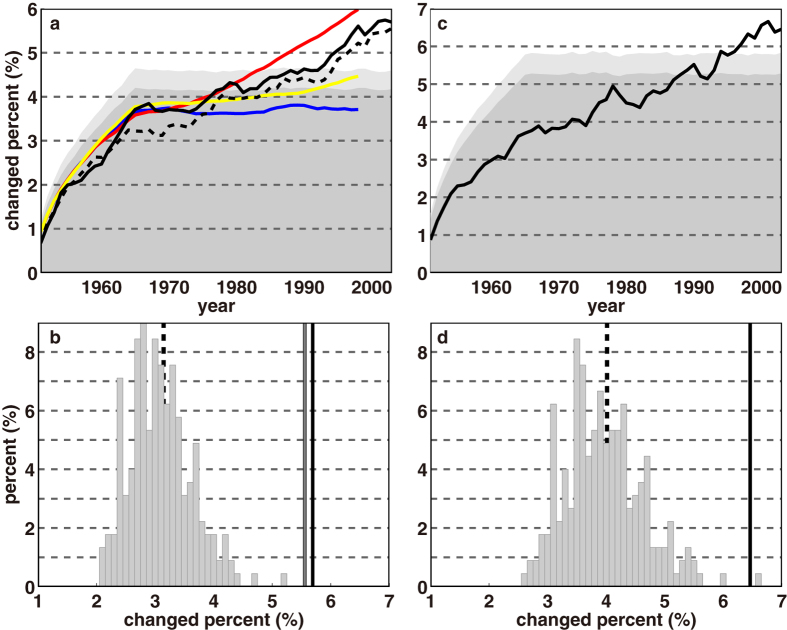
(**a**) Percentage of world land area with a climate type change in each year compared to 1950 from UD (black solid line) and CRU (black dashed line) observations, and HIST-ALL (yellow), HIST-GHG (red), and HIST-NAT (blue) runs. Dark shading shows the 95^th^ percentile of changed climate types relative to the starting year based on 54-year samples of PI-CTL simulations as illustrated in (**b**), and light shading shows the estimated 95^th^ percentile if the variance is doubled. (**b**) Distribution of 54-year changed area percentage due to internal climate variability based on 225 PI-CTL samples (gray bars) with their mean value of 3.1% indicated by the black dashed line. The vertical solid lines are the observed percentages of global land area with a changed climate class in the UD dataset (about 5.7%, black line) and the CRU dataset (about 5.6%, gray line). (**c**) Same as (**a**) but for the GISS dataset. (**d**) Same as (**b**) but for the GISS dataset. Panel (**d**) uses the same PI-CTL runs as in (**c**) but grids are averaged at the GISS resolution and calculations exclude GISS “missing” grid boxes.

For any year of the 54-yr period, the percentage of the world land area that has experienced a major climate type change compared to 1950 in observations or the first year of a PI-CTL simulation can be calculated. Figure 1a shows the evolution of this percentage from 1951–2003 in observations (black solid line) and the 95^th^ percentile from 225 54-yr PI-CTL simulations (dark gray shading). Because each year is the middle year of a 15-year sample, the percentage of land area with a changed climate type increases rapidly in the first 15 years (as the overlap of 15-year periods diminishes so 1966, or the 1958–1972 average, is the first period with no overlap with 1950, or the 1943–1957 average), then stabilizes (for 95^th^ percentile shading or blue line, because the mean has negligible change), or rises slowly (for other lines, because the mean climate has changed). From Fig. 1b, based on these control simulations, natural variability would usually cause about 2–4% of the global land area to have a different major climate type than 54 years earlier. In Fig. 1a, the 95^th^ percentile of land area with a different major climate type stabilizes at 4.1–4.2% for all time separations exceeding the 15-yr averaging period in control runs, so the distribution as shown in Fig. 1b for 54-year separations is very similar for any time separation >15 years. However, based on observations, the area with major climate type changes becomes consistently greater than zero at the 5% significance level beginning around 1980. This suggests that significant climate shifts were detectable before the recent dramatic and accelerated warming.

The geographical distribution of observed (1950–2003, detrended) and CMIP5 model control run variances of grid box annual averaged surface air temperature (SAT) and precipitation over land is shown in Supplementary Fig. S1. The general features of observed variability are well simulated in the multi-model ensemble-mean simulations. The CMIP5 models have comparable or larger variability of temperature and precipitation than observed over 86% of land grid boxes (excluding the Antarctic). Therefore, the significance of the change in Fig. 1a is not likely overestimated. However, a more conservative test of doubling the variance of distribution of 54-year changed area percentage due to internal climate variability in Fig. 1b gives an alternative 95% cumulative probability in Fig. 1a shown by light gray shading, and this level is consistently exceeded starting 1996. About 5.7% of the land surface has experienced shifts in major climate types by 2003, and changes are scattered in all major types rather than being constrained in only one or two.

**Figure 2:**
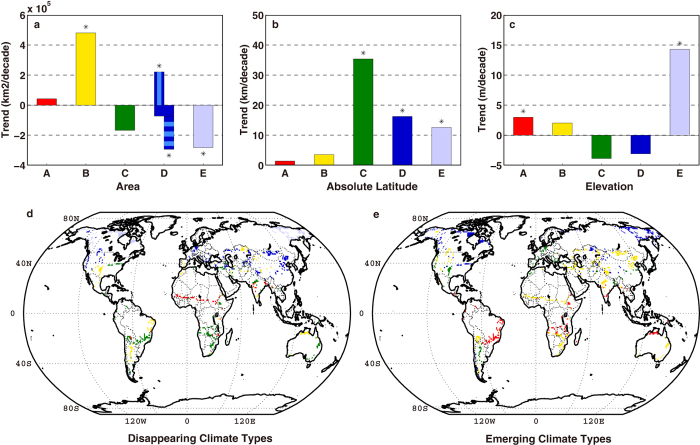
(**a**) Linear trends in areas of 5 major climate types for 1950–2003 using the UD dataset; asterisks denote significant trends at the 5% level. A positive trend of high-latitude (north of 55°N) D climate and a negative midlatitude (south of 55°N) D climate are over-plotted in blue with the net negative trend of D climate in dark blue. (**b**) and (**c**) are the same as (**a**), but for trends in average absolute latitude (positive indicates poleward) and elevation, respectively. (**d**) Map showing grid boxes with a major climate type in 1950 that “disappeared” (changed to another type) by 2003. Grid boxes are 1° × 1°. Colors are the same as in upper panels. (**e**) is the same as (**d**), but for “emerging” climate types (by 2003) in the same grid boxes. We generate five sub-panels (**a–e**) using Matlab software, and integrate them into this figure using Adobe Illustrator.

Figure 2 shows linear trends of area, latitude and elevation indices from 1950 to 2003. Expansion (shrinkage) exceeding 5% significance is found in arid (polar) climate at a rate of 4.8 × 10^5^ (−2.8 × 10^5^) km^2^ decade^−1^. Significant poleward shifts are detected in temperate, continental and polar climate averaging 35.4, 16.2 and 12.6 km decade^−1^, respectively, and significant elevation shifts in tropical and polar climate averaging 3.0 and 14.3 m decade^−1^, respectively. Trends of total areas and averaged elevations of temperate and continental climate are negative and not significant, but both shrinkage of continental climate over regions south of 55°N (−2.9 × 10^5 ^km^2^ decade^−1^) and expansion of continental climate north of 55°N (2.2 × 10^5^ km^2^ decade^−1^) are statistically significant. All climate types show net poleward movement (Fig. 2b) due to poleward expansion of A and B climates and poleward shrinkage of C, D, and E climates. Even if the estimated variance of each trend is doubled in Fig. 2, the above significant trends can still be detected at the 5% level.

Figure 2d,e show grid boxes with disappearing and emerging climate types from 1950–2003. The most conspicuous feature is a worldwide expansion of B climate (mainly semiarid) at the expense of C and midlatitude D climate. Rising temperature and decreasing precipitation are about equally important in causing the expansion of semiarid climate in Asia and western North America, while the contribution of decreasing precipitation to the increasing semiarid climate is much larger than that of temperature over North Africa, South Africa and South America. Overall, temperature and precipitation play similar roles in the expansion of B climate (Supplementary Figs S2d–e). In the tropics, B replaces A climate over northern India and the southern Sahara due to reduced precipitation, but A climate emerges in southern India and higher elevations over South America, northern South Africa and northern Australia mainly due to increasing temperature. The above changes raise the average elevation of A climate. Over the low level regions north of 55°N, the current E climate distribution is more likely to be affected first by increasing warming, but higher elevations remain cold enough to maintain the existing climate zones. The replacement of E by D climate is found over Alaska, northern Canada, Siberia and Far East regions in Asia, and over the Tibetan Plateau, leading to a significant shrinkage and higher elevation of E climate, and a poleward shift of D and E climate. The rising elevation of A and E climates was reported in a modeling study^19^. The reduction of the area of C climate is mainly caused by the shift to A or B climate over large regions of South Africa and South America driven by both temperature and precipitation changes. Both shifts of D to C climate in large areas over Europe and East Asia due to increasing temperature and B to C climate over South America due to increasing precipitation contribute to a significant poleward shift of C climate.

Changes in climate types are generally not seen in interpolated or “missing” boxes, which are mostly in the Sahara, South Africa, Mid-East, Southeast Asia, northern South America, Greenland and Antarctica (Figs S2d–e). Most land areas with no weather station within 1200 km are extremely dry or cold (or in a tropical rain forest), and the climate is not close to a major type threshold. Therefore, results of significant changes of the land area percentage index and major climate indices in Figs 1, 2 are not dependent on the interpolated grid boxes.

### Sensitiveness to different observed datasets

The UD and CRU datasets produce very similar climate type change detection results (Table 1). The percentage of the world land area that has experienced a major climate type change compared to 1950 has an almost identical trend in both datasets (Fig. 1a,b). Expansion exceeding 5% significance is found in the areas of arid climate and continental climate north of 55°N at a rate of 4.2 × 10^5^ and 2.3 × 10^5^ km^2^ decade^−1^ respectively, while significant shrinkage of polar climate and continental climate south of 55°N is found at −2.9 × 10^5^ and −3.2 × 10^5^ km^2^ decade^−1^, respectively. Significant poleward shifts are detected in temperate, continental and polar climate averaging 45.6, 17.1 and 9.8 km decade^−1^, respectively. Significant elevation shifts in tropical and polar climate are also detected averaging 3.1 and 17.6 m decade^−1^, respectively. The above changes and similar results of grid boxes with disappearing and emerging climate types from 1950–2003 for the CRU dataset are shown in Supplementary Fig. S3.

The main findings are also reproduced using the GISS dataset (Table 1). Differences result partly from the larger grid box size than with the other data sets, and because “missing” grid boxes are excluded from the numerator and denominator of calculations. Figure 1c–d show the percentage of land area experiencing changes in major climate classes since 1950 based on the GISS dataset. The percent of area with a climate type change has exceeded the 5% significance level since the early 1990 s. About 6.5% of the global land surface has experienced shifts in major climate types by 2003, which is significantly greater than zero at the 5% significance level even if the variance of distribution of 54-year changed area percentage due to internal climate variability in Fig. 1d is doubled.

Changes in three indices of area, absolute latitude and elevation are listed in Table 1 and displayed in Supplementary Fig. S4 for the GISS dataset. The main results do not change noticeably for B-Area, E-Area, C-Latitude and D-Latitude. Trends of E-Latitude and E-Elevation are still significant, but increases in magnitudes are larger than for the UD dataset due to many “empty” grid boxes over the Antarctic and Greenland (Figs S4d–e). The trend of A-Elevation is not significant, which can still be explained by “empty” grid boxes over large areas of Brazil and Angola, while in the UD dataset, these areas contribute to the growth of A-Elevation. The emerging climate map based on the GISS dataset (Fig. S4e) is almost identical to that from the UD dataset (Fig. 2e).

These sensitivity test results suggest that significant changes in major climate types are robust and consistent among different datasets, so we use the UD dataset in the following attribution study.

### Attribution of significant changes of climate type indices

To determine possible roles of external anthropogenic and natural radiative forcings in the above climate shifts, the four indices are calculated from the multi-model averaged historical CMIP5 simulations forced by observed atmospheric composition changes (including anthropogenic forcings such as greenhouse gases and sulfate aerosols and natural forcings such as volcanic eruptions and solar output changes, termed HIST-ALL), by greenhouse gas forcings only (HIST-GHG), or by natural forcings only (HIST-NAT). Supplementary Table S1 lists the selected model runs^25^, which use historical data ending 2005 (the last 15-year average is centered on 1998). Figure 1a shows multi-model ensemble means of the percentage of the world land area with a climate type change from 1950. By 1998, about 4.5%, 6.0% and 3.7% of the land surface has experienced shifts in major climate types in HIST-ALL, HIST-GHG and HIST-NAT simulations, respectively. Both HIST-ALL and HIST-GHG simulations fairly well reproduce the broad scale pattern of observed temperature induced climate type changes in the UD dataset (Supplementary Figs S2f–g), including emergence of tropical climate over South-Southeast Asia, southeast Africa, Northwest tip of South America and the Southern Hemisphere, expansion of B climate in the midlatitude Northern Hemisphere, and a shift of E to D climate in high-latitudes.

In Fig. 1a,c, as discussed before, all curves show a rapid rise in the percentage of the world land area that has experienced a major climate type change until 1966 due to diminishing overlap of 15-year periods with 1950 (1943–1957), followed by either little or no consistent change, or a gradual rise. In 1966, the observations and all simulation averages have changes in major climate types from 1950 in about 3.7% of the global land area. This slightly exceeds the mean (~3.1%) of a distribution as in Fig. 1b (which reflects average model-generated natural variability), but any trend included in this period does not reach 95% statistical significance.

**Figure 3:**
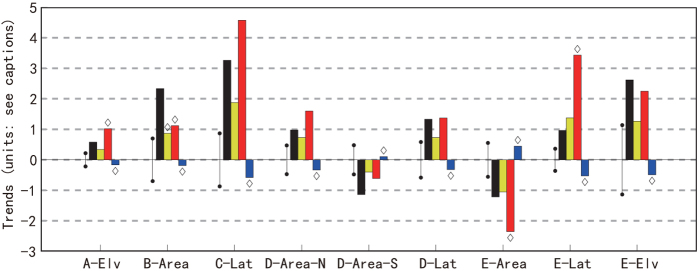
Significant observed trends (black bars) marked with * in Fig. 2 **and the corresponding simulated trends of indices for 1950–1998; yellow, red and blue bars denote HIST-ALL, HIST-GHG and HIST-NAT runs, respectively.** Each error bar at the left of an observed trend is the standard deviation (σ) of such trend estimated from 225 samples of 54-yr CMIP5 control runs and represents the natural variability of the observed or modeled trend. Simulated trends significantly different from the observation at the 5% level are marked with diamonds. The units are 2 × 10^5 ^m^2^ decade^−1^ for area, 10 km decade^−1^ for latitude and 5 m decade^−1^ for elevation indices.

For significant trends in Fig. 2, Fig. 3 shows corresponding trends for HIST-ALL, HIST-GHG, and HIST-NAT multi-model averages. For each major climate type, the HIST-ALL and HIST-GHG experiments qualitatively reproduce all significant observed trends. A two-sided consistency test is conducted to determine whether the difference between the observed and any simulated trend is significantly different from zero at the 90% confidence level for each index of major climate type. The observed trends are consistent with those in the HIST-ALL run, except that the simulated B climate expansion is smaller than observed, which is explained by the finding that the models underestimate the observed precipitation trends^8^,^26^. By consistent, we mean that the observed trend lies within the 90% confidence interval obtained by combining the uncertainty for the ensemble-mean forced model trend with the uncertainty estimated from control runs. HIST-NAT trends are small and have the opposite sign of all significant observed trends. In Fig. 3, increases in well mixed greenhouse gases (based on HIST-GHG) are the main driver of significant changes in major climate types, but HIST-GHG runs overstate most trends because they omit offsetting cooling factors such as sulfate aerosols.

### Projections of changes of climate type indices

The increase of arid and tropical climates squeezes the areas occupied by C and D climates in the subtropics and midlatitudes. Although observations show insignificant contraction in C climate and expansion of A climate before 2010, projections (Supplementary Fig. S5) indicate acceleration of changes of areas for these two major climates after about 2006 to a significant level by 2020, while significant expansion of B and shrinkage of midlatitude (south of 55°N) D climates continue under both the RCP 4.5 and RCP 8.5 scenarios. Associated with the projected expansions of A and B climates, trends in average absolute latitude of these two climate types are projected to reach statistical significance by 2020. The poleward shifts in C and E climates are projected to accelerate in the next decades. Significant average elevation increases in tropical and polar climates are projected to continue by 2100, while the decreasing average elevation of the C climate is expected to reach statistical significance around 2020. These results suggest that the projected future temperature and precipitation changes are able to generate new emerging significant shifts in global major climate regimes. Some of these anthropogenic-induced emerging signals of climate types will be detected in the next decade.

## Conclusions

Previous detection and attribution results are strengthened by the finding that changes in major Köppen climate types since 1950 have occurred worldwide and are almost entirely attributed to the observed anthropogenic increase in greenhouse gas concentrations. Model runs project accelerating anthropogenic-induced major climate type changes in the next decades. As the Köppen climate classification links the Earth’s climates with the qualitative features of the vegetation, results here indicate that observed climate changes might already be causing significant impacts on vegetation in areas where the major climate class has changed, and model projections imply increasing future impacts.

## Methods

This study uses updated thresholds^11^ for five major Köppen climate classes (30 subtypes are not of concern here) based on climate data at a location, with tests performed in the following order to resolve conflicts: Arid (class B; MAP (mean annual precipitation, mm) <10 × P_threshold_, where the aridity threshold P_threshold _= 2 × MAT (mean annual temperature, °C) if 70% of MAP occurs in winter; and P_threshold _= 2 × MAT + 28 (mm) if 70% of MAP occurs in summer; otherwise P_threshold _= 2 × MAT + 14 mm), Tropical (class A; T_cold_ (temperature of the coldest month) ≥18 °C), Polar (class E; T_hot_ (temperature of the hottest month) < 10 °C), Temperate (class C; T_hot _* > *10 °C and 0 *< *T_cold _* < *18 °C), Continental (class D; T_hot _* > *10 °C and T_cold _ ≤ 0 °C).

For detection, attribution, and projection studies, monthly temperature and precipitation grids are downloaded from CMIP5 model runs^25^ using PI-CTL, HIST-ALL, HIST-GHG, HIST-NAT, RCP4.5, and RCP8.5 scenarios (Supplementary Table S1). Years are arbitrary for PI-CTL runs. HIST-ALL runs are driven by annual forcing values reconstructed from observed data (such as greenhouse gas concentrations and actual volcanic eruptions), and this study downloads 1940–2005 monthly grids for runs based on all forcing factors (HIST-ALL), greenhouse gases only (HIST-GHG), and natural factors only (HIST-NAT). RCP runs have specified annual forcings projected for 2006–2100, and each run is initiated from a HIST-ALL run.

Grid data pretreatment involves four steps: (1) The UD and CRU observational datasets and model outputs are regridded onto a 1° × 1° grid to guarantee the same resolution, while the UD precipitation is also regridded onto a 2° × 2° grid to match the temperature in the GISS dataset. (2) GISS monthly grid box anomalies minus the corresponding monthly averaged anomaly for 1940–1960 are first computed, and are then added to the observational UD 1940–1960 grid box monthly climatology to produce monthly grid box temperature. (3) For the historical period (1940–2005) of the CMIP5 runs, anomalies relative to monthly climatology for 1940–1960 are similarly computed and are then added to the observational UD 1940–1960 monthly means. For the prediction period (2006–2100) of the CMIP5 runs, anomalies relative to a 15-year climatology (historical for 1996–2005 and RCP for 2006–2010) are added to the observational 1996–2010 UD monthly means. These downscaling steps ensure the consistency between observation and simulations. This is necessary since the Köppen-Geiger scheme is quite sensitive to thresholds and models have problems in simulating the present-day distribution. (4) Third, a 15-yr running smooth is applied to all variables in both observation (including the UD, CRU and GISS) and model (including PI-CTL) data to reduce the probabilistic influence of shorter-term climate variability. The 15-year averaging period is chosen as an optimal smoothing interval for Köppen or related classifications^12^, but results here are not highly sensitive to the length of the averaging period. There is a 7 year loss on each side of the time interval, so the 1950 starting year represents the 1943–1957 average. Analysis periods end with 2003 (1996–2010) for observations, 1998 (1991–2005) for historical runs and 2093 (2086–2100) for RCP runs.

The major climate zones (A - tropical, B - arid, C - temperate, D - cold, and E - polar) are then defined using the Köppen-Geiger scheme with updated criteria^11^. If tests are applied in the order B, A, E, C, D, simplified criteria as stated at the beginning of this section will always assign a unique climate class. Three indices are then computed to depict changes in climate regions. For each major climate type, the area index is the total area with that climate type, the latitude index is the global averaged absolute latitude (an increase is a poleward average movement of the climate type), and the elevation index is the average altitude. Since one climate type is assigned to each 1 × 1° land grid box (111 × 111 km at the equator), all grid boxes are weighted by the cosine of their latitude to ensure that equal areas are afforded equal weight. In Fig. 2b, the average latitude trend (° decade^−1^) is multiplied by 111 to be expressed as km decade^−1^.

To test whether an observed change/trend is significantly larger than internal variability, model outputs from pre-industrial control (PI-CTL) runs (pretreated as above, with 15-year averaging) are used to estimate the standard deviation (σ) of each change/trend in a naturally fluctuating climate. Steps of estimation are as follows. (1) Major climate types are computed for each PI-CTL run. (2) A 54-year sample is taken every 54 years from the first year in each model run, giving a total of 225 sample time series. (3) For each sample, we calculate the percentage of the land surface experiencing climate shifts compared to the first year, as well as linear trends of the other three indices (changes in area, average absolute latitude, and average elevation of each climate type). Figure 1b shows the distribution of percent of land area with changed major climate types with 54-year time separation (from the first to the last year of each sample) for the 225 samples, and the darker gray shading in Fig. 1a shows the 95^th^ percentile of percentages of land area with changed major climate types with time separations from 1 to 54 years. A change is significant with 95% confidence if the percentage of changed climate types exceeds the 95^th^ percentile. For the other indices, a 1-sided significance test is used since trends of these variables generally follow normal distributions. An observed trend (OBS) is statistically significant at the 5% level if 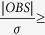 1.96. (4) For Supplementary Fig. S5, the estimated internal variability (standard deviation) for each variable for trends starting in 1950 and ending in all years through 2093 is obtained by following the same procedure with PI-CTL samples of length 2 to 144 years, and the 95% confidence range is shown by gray shading in each inset panel. (5) For Fig. 3, the natural variability of the observed or modeled trend (the standard deviation, σ) is estimated from 225 samples of 54-yr CMIP5 control runs. We conduct a two-side consistency test to determine whether the difference between the observed and any simulated trend (|difference|) is significantly different from zero at the 90% confidence level at each region and grid box (|difference| ≥ 1.64σ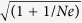 ), where we assume that observed and simulated trends from the control runs are approximately normally distributed with the same standard deviation (σ), and *Ne* is the number of ensemble members in the last row of Supplementary Table S1. The forced trend of an index of climate type change is not significantly different from the observed trend if the observed trend lies within the 90% confidence interval obtained by combining the uncertainty for the ensemble-mean forced model trend with the uncertainty estimated from control runs.

The observed annual variance of the detrended surface temperatures and precipitation is compared with the variance in the control simulations (Supplementary Fig. S1) to evaluate the quality of the simulations of natural internal climate variability. Simple linear detrending is used to attempt to remove some of the possible anthropogenic signal in the observations. The variances of the detrended observed and control simulated temperatures of each model are calculated at each of the grid boxes. An F-test is utilized to determine whether the multi-model mean of simulated variance in control runs is significantly larger or smaller than the corresponding observed variance at each grid box at the 5% significance level.

## Additional Information

**How to cite this article**: Chan, D. and Wu, Q. Significant anthropogenic-induced changes of climate classes since 1950. *Sci. Rep.* **5**, 13487; doi: 10.1038/srep13487 (2015).

## Supplementary Material

Supplementary Information

## Figures and Tables

**Table 1:** Linear trends in total area occupied by each major climate type, average absolute latitude of each major climate type; and averaged elevation of each major climate type in the UD, CRU and GISS datasets.

	A	B	C	D	D-North	D-South	E
Area	0.4	**4.8**	−1.7	−0.7	**2.2**	**−2.9**	**−2.8**
Latitude	1.4	3.5	**35.4**	**16.2**			**12.6**
Elevation	**3.0**	2.0	−3.8	−3.1			**14.3**
CRU							
Area	0.8	**4.2**	−1.2	−0.9	**2.3**	**−3.2**	**−2.9**
Latitude	4.1	2.8	**45.6**	**17.1**			**9.8**
Elevation	**3.1**	−0.1	−6.6	0.0			**17.6**
GISS (with missing boxes excluded from calculation)							
Area	0.2	**4.5**	−0.6	−0.9	**2.5**	**−3.4**	**−3.1**
Latitude	2.6	4.8	**32.4**	**19.4**			**31.6**
Elevation	2.4	0.5	−2.9	−2.1			**24.1**

Significant trends of these indices at the 5% statistical significance level are in bold. Units are 10^5^ km^2^ per decade for area, km per decade for latitude, and m per decade for elevation.
