# Supplementary material for: Significant anthropogenic-induced changes of climate classes since 1950
Source: Sci Rep. 2015 Aug 28;5:13487. doi: 10.1038/srep13487 (PMC4551970; doi:10.1038/srep13487)
Supplement: Supplementary Information [file srep13487-s1.pdf]

# Significant anthropogenic-induced changes of climate classes since 1950

(Supplementary Information)

Duo Chan[1] and Qigang Wu[1*]

[1]School of Atmospheric Science, Nanjing University, Hankou Road #22, Nanjing, Jiangsu, China, 210093

* Correspondence to: qigangwu@nju.edu.cn

May, 19, 2015

**Supplementary Table S1.** CMIP5 model runs used in this study. Numbers are years of length of the preindustrial control (PI-CTL) run, and number of ensemble members for historical runs with all forcings (HIST-ALL), historical with greenhouse gas forcing (HIST-GHG), historical with natural forcing (HIST-NAT), and RCP4.5 and RCP8.5 (radiative forcing of 4.5 or 8.5 W $m^{-2}$ projected in 2100 relative to preindustrial) experiments.

| MODELS | PI-CTL (years) | HIST-ALL | HIST-GHG | HIST-NAT | RCP4.5 | RCP8.5 |
|---|---|---|---|---|---|---|
| ACCESS1.0 | 500 | 2 | -- | -- | 1 | 1 |
| ACCESS1.3 | 500 | 3 | 2 | -- | 1 | 1 |
| BCC-CSM1.1 | 500 | 3 | 1 | 1 | 1 | 1 |
| BCC-CSM1.1(m) | 400 | 3 | -- | -- | 1 | 1 |
| CanESM2 | 700 | 5 | 5 | 5 | 5 | 5 |
| CCSM4 | 500 | 5 | 3 | 4 | 5 | 5 |
| CESM1-CAM5 | 300 | 3 | -- | -- | 3 | 3 |
| CMCC-CESM | 200 | 1 | -- | -- | -- | 1 |
| CMCC-CM | 200 | 1 | -- | -- | 1 | 1 |
| CNRM-CM5 | 500 | 5 | 5 | 5 | 1 | 3 |
| CSIRO-Mk3.6.0 | 500 | 5 | 5 | 5 | 5 | 5 |
| FGOALS-g2 | -- | 5 | 1 | 3 | 1 | 1 |
| FIO-ESM | -- | 3 | -- | -- | 1 | 1 |
| GFDL-CM3 | 500 | 5 | 3 | 3 | 1 | 1 |
| GFDL-ESM2G | 500 | 1 | -- | -- | 1 | 1 |
| GFDL-ESM2M | 500 | 1 | 1 | 1 | 1 | 1 |
| GISS-E2-H | 500 | 5 | 5 | 5 | 5 | 2 |
| GISS-E2-R | 500 | 5 | 5 | 5 | 5 | 2 |
| HadGEM2-AO | -- | 1 | -- | -- | 1 | 1 |
| HadGEM2-CC | -- | 1 | -- | -- | 1 | 1 |
| HadGEM2-ES | 300 | 5 | 4 | 4 | 4 | 4 |
| INM-CM4 | -- | 1 | -- | -- | 1 | 1 |
| IPSL-CM5A-LR | 1000 | 5 | 3 | 3 | 4 | 4 |
| IPSL-CM5A-MR | 300 | 3 | 3 | 3 | 1 | 1 |
| IPSL-CM5B-LR | 300 | 1 | -- | -- | 1 | 1 |
| MIROC-ESM | 500 | 3 | 3 | 3 | 1 | 1 |
| MIROC-ESM-CHEM | 200 | 1 | 1 | 1 | 1 | 1 |
| MIROC5 | 600 | 5 | -- | -- | 3 | 3 |
| MPI-ESM-LR | 1000 | 3 | -- | -- | 3 | 3 |
| MPI-ESM-MR | 1000 | 3 | -- | -- | 3 | 1 |
| MRI-CGCM3 | 500 | 3 | 1 | 1 | 1 | 1 |
| NorESM1-M | 500 | 3 | 1 | 1 | 1 | 1 |
| SUM | 13500 | 99 | 52 | 53 | 65 | 60 |

## Supplementary Figure Legends

**Supplementary Figure S1.** The variances of annual mean SAT ($K^2$)and Precipitation ($mm^2$) in observations (a, b) and the CMIP5 multi-model control simulations (c, d). Black/White shadings mark grid boxes with simulated variance significantly larger/smaller than observation at 5% significance level in the F-test. The spatial correlation coefficient between the observed and modeled standard deviation fields is 0.82 and 0.76 for temperature and precipitation, respectively. The CMIP5 model has significantly larger, smaller and comparable variability of temperature (precipitation) than observed over 20.7% (31.2%), 10.7% (14.8%), 68.6% (54.0%) of global land grid boxes (excluding the Antarctic). We generate four sub-panels (a-d) using Matlab software, and integrate them into this figure using Adobe Illustrator.

**Supplementary Figure S2.** Analysis of factors causing changes in climate types in the historical period from 1950 to 2003 using observed data (**a** to **e**) or to 1998 using HIST-ALL (**f**) or HIST-GHG (**g**) model runs, with each stated year representing a 15-year centered average. (**a** to **c**) Same as in Fig. 2 with the left bar for each climate type the same as in Fig. 2, but the middle (red edge) bars are temperature-induced changes (climate types derived with changing temperature and 1950 precipitation values), and the right (blue edge) bars are precipitation-induced changes (changing precipitation and 1950 temperature values). An asterisk denotes that a trend is significant at the 95% confidence level. (**d**) As in Fig. **2e**, showing grid boxes with changed 2003 climate types when precipitation is held at 1950 values. (**e**) As in Fig.

**2e**, showing changed 2003 climate types when temperature is held at 1950 values. (**f** and **g**) Changed grid box climate types in 1998 from 1950 using multi-model averages of HIST (**f**) and HIST-GHG (**g**) runs. We generate seven sub-panels (a-g) using Matlab software, and integrate them into this figure using Adobe Illustrator.

**Supplementary Figure S3. Same as Figure 2 but for the** CRU **dataset.** Grid boxes are 1 °× 1 °. We generate five sub-panels (a-e) using Matlab software, and integrate them into this figure using Adobe Illustrator.

**Supplementary Figure S4. Same as Figure 2 but for the** GISS **dataset.** Grid boxes are 2 °× 2 °, and the GISS dataset omits boxes (shown in gray) with no stations within 250 km. We generate five sub-panels (a-e) using Matlab software, and integrate them into this figure using Adobe Illustrator.

**Supplementary Figure S5.** Temporal evolution of each index and each major climate type, for the historical period (1950-2003) and projections (2003-2093). In the historical period, the solid black line is the UD observation; the dashed black line, solid red line and solid blue line denote multi-model averaged indices derived from HIST-ALL, HIST-GHG and HIST-NAT experiments, respectively. In the projection period, the solid orange and magenta lines are ensemble mean indices from RCP8.5 and RCP4.5 scenarios. Light orange and magenta shadings are corresponding estimated 95% cross-model uncertainties. Units are $km^2$, degrees ( °) and m for area, absolute latitude and elevation indices. The inset figure in each panel is the temporal evolution of the linear trend (units $decade^{-1}$) since 1950 over the entire observation

and projection period. Orange and magenta lines denote RCP8.5 and RCP4.5 predictions, respectively. Gray shadings are estimated range of long-term linear trends in a natural fluctuating climate as a function of time span at the 95% confidence level. We generate all sub-panels using Matlab software, and integrate them into this figure using Adobe Illustrator.

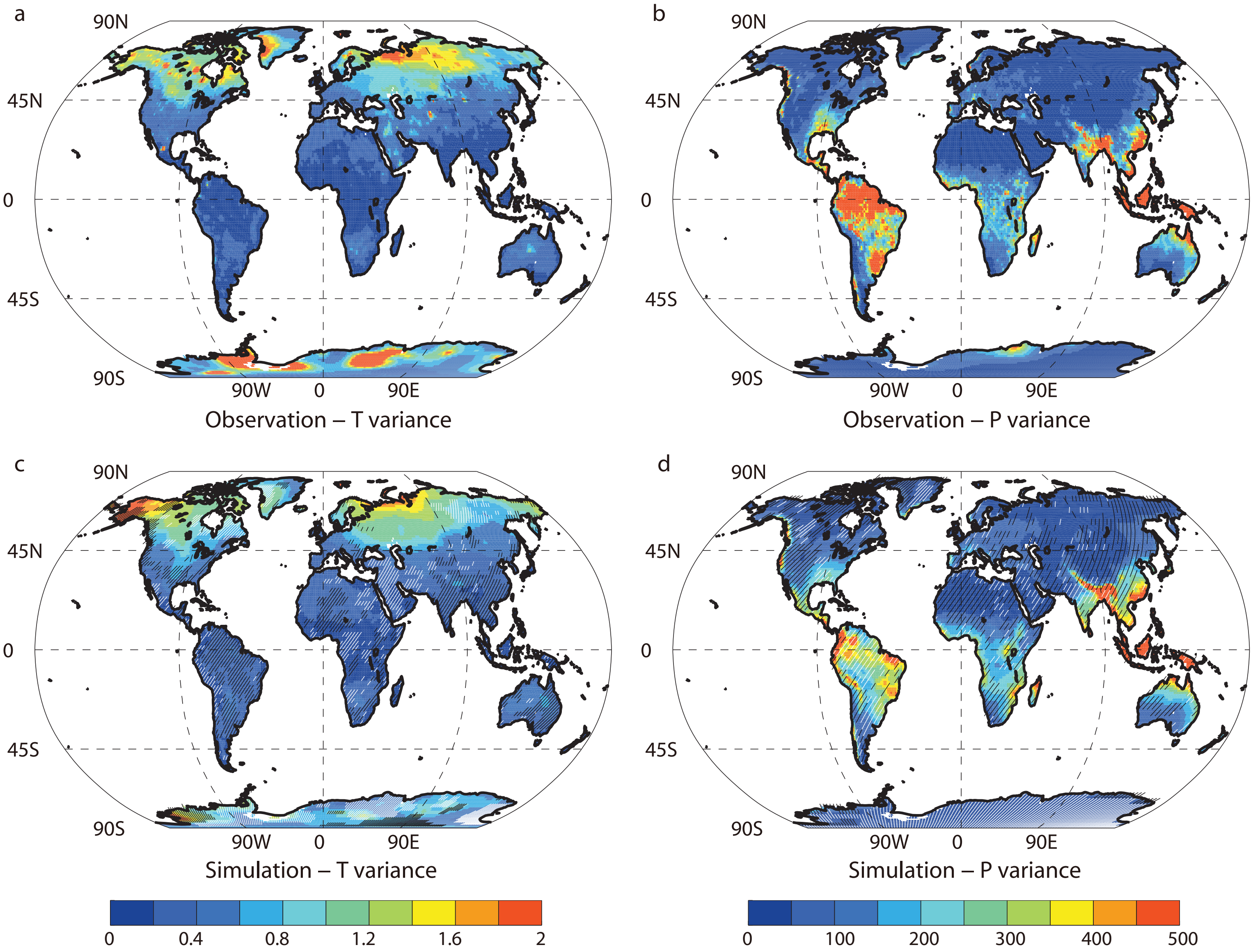
a
90N
45N
0
45S
90S
90W
0
90E
Observation – T variance
b
90N
45N
0
45S
90S
90W
0
90E
Observation – P variance
c
90N
45N
0
45S
90S
90W
0
90E
Simulation – T variance
d
90N
45N
0
45S
90S
90W
0
90E
Simulation – P variance
0
0.4
0.8
1.2
1.6
2
0
100
200
300
400
500

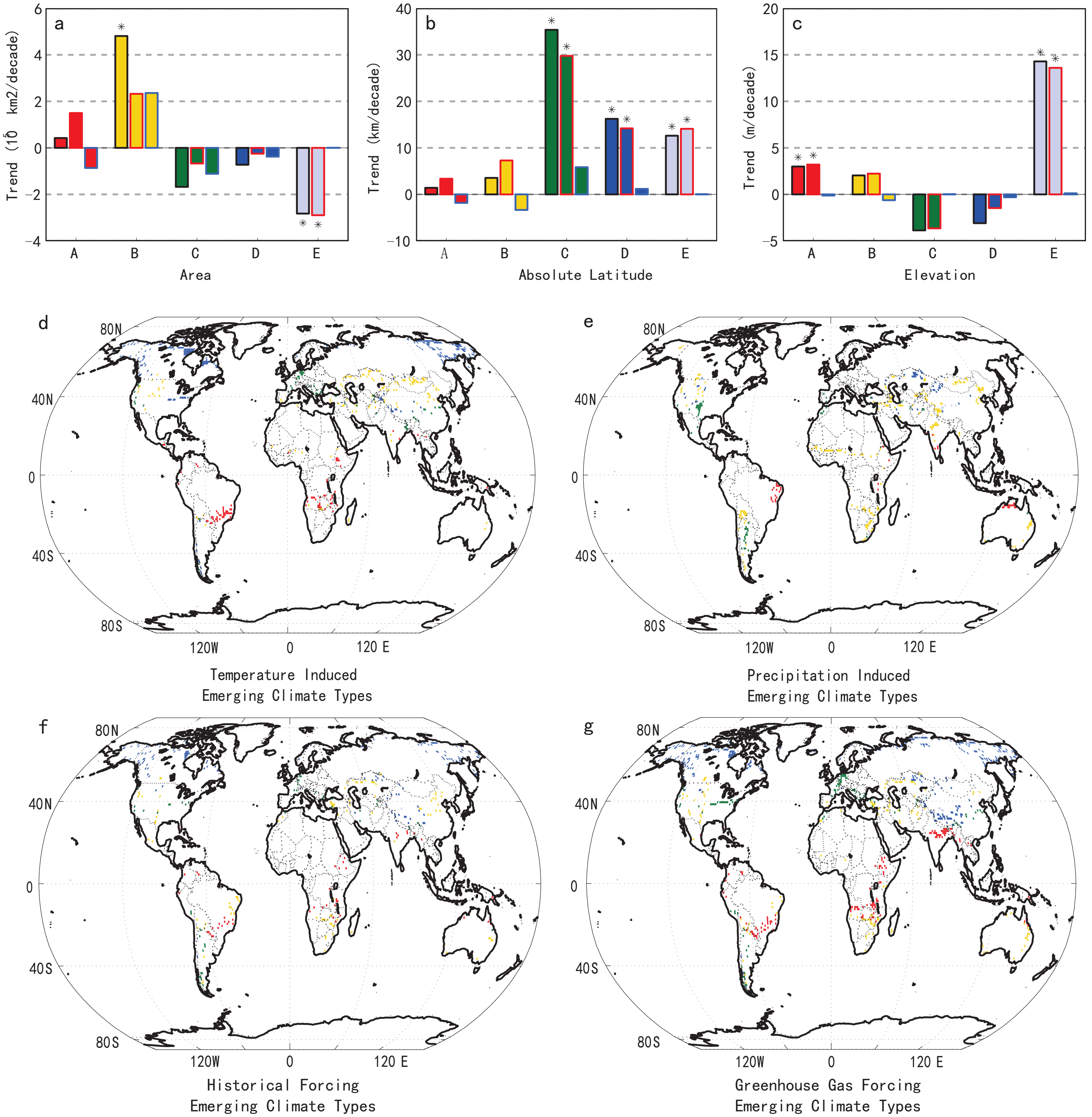
a
Trend (10 km2/decade)
6
4
2
0
−2
−4
A
B
C
D
E
Area
b
Trend (km/decade)
40
30
20
10
0
−10
Absolute Latitude
c
Trend (m/decade)
20
15
10
5
0
−5
Elevation
d
80N
40N
0
40S
80S
120W
120 E
Temperature Induced
Emerging Climate Types
e
Precipitation Induced
Emerging Climate Types
f
Historical Forcing
Emerging Climate Types
g
Greenhouse Gas Forcing
Emerging Climate Types

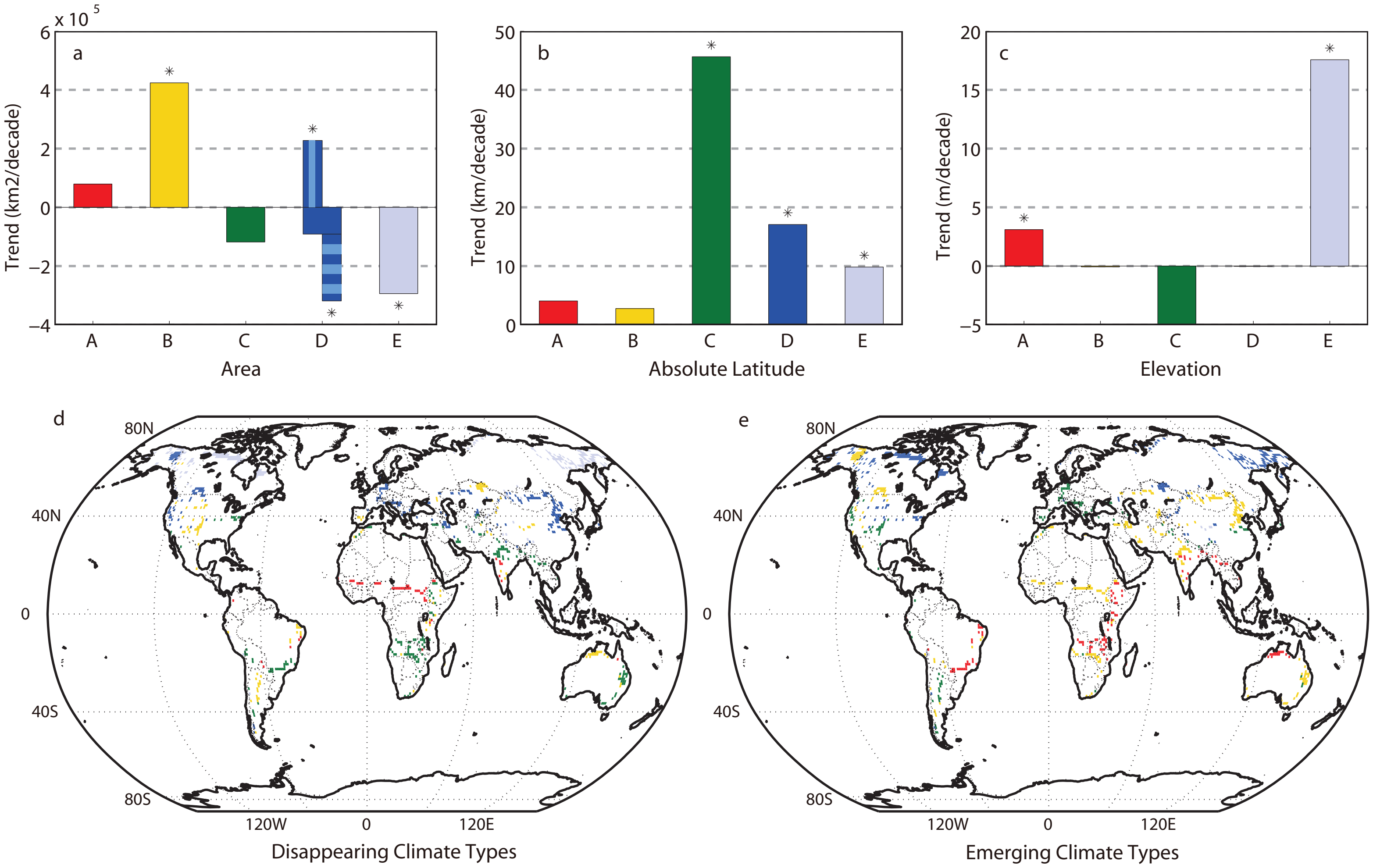
x 10^5
a
Trend (km2/decade)
A B C D E
Area
b
Trend (km/decade)
Absolute Latitude
c
Trend (m/decade)
Elevation
d
80N
40N
0
40S
80S
120W 0 120E
Disappearing Climate Types
e
Emerging Climate Types

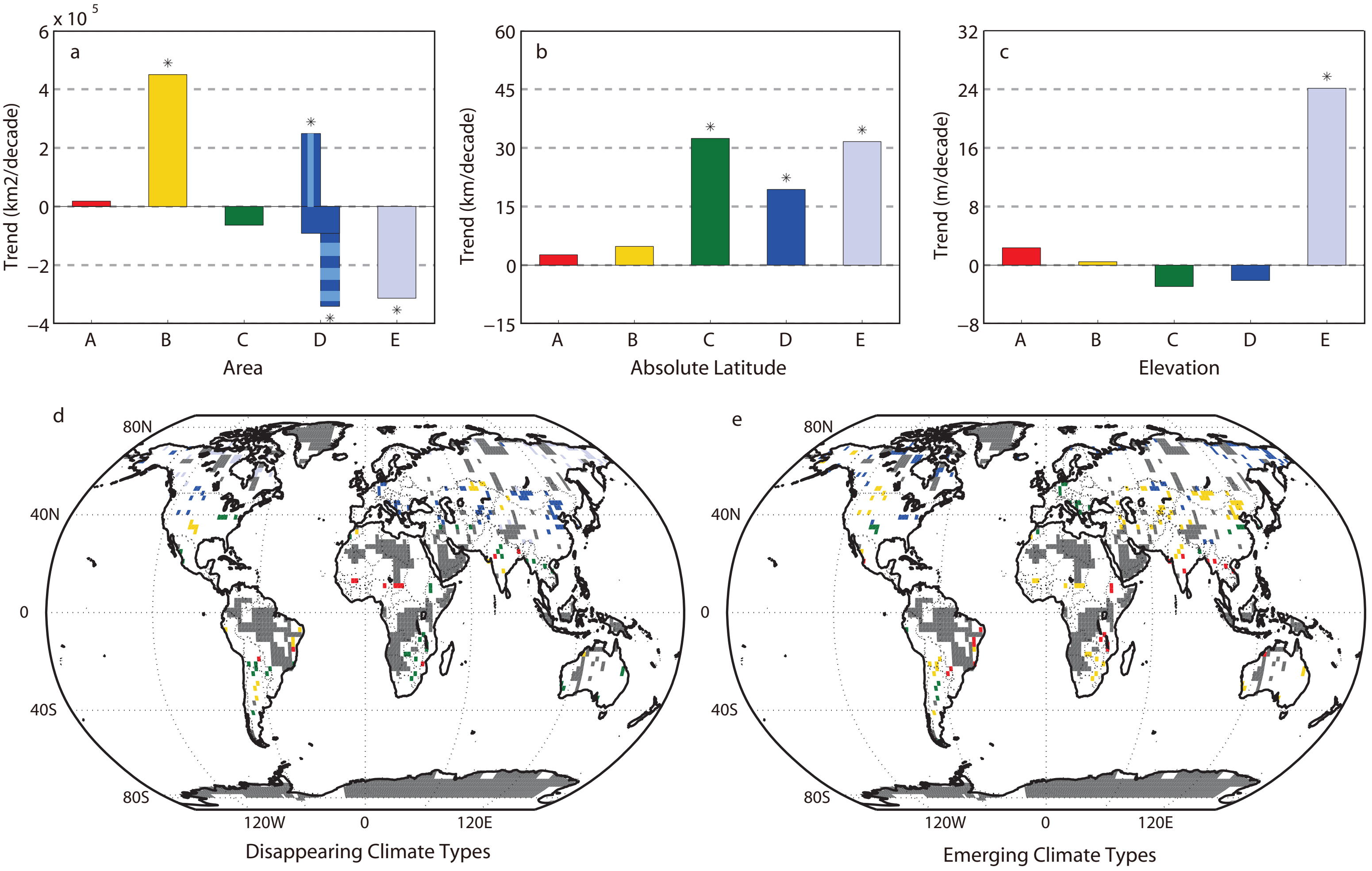

x 10^5
Trend (km2/decade)
a
Area
b
Trend (km/decade)
Absolute Latitude
c
Trend (m/decade)
Elevation
A
B
C
D
E
d
Disappearing Climate Types
e
Emerging Climate Types
80N
40N
0
40S
80S
120W
120E

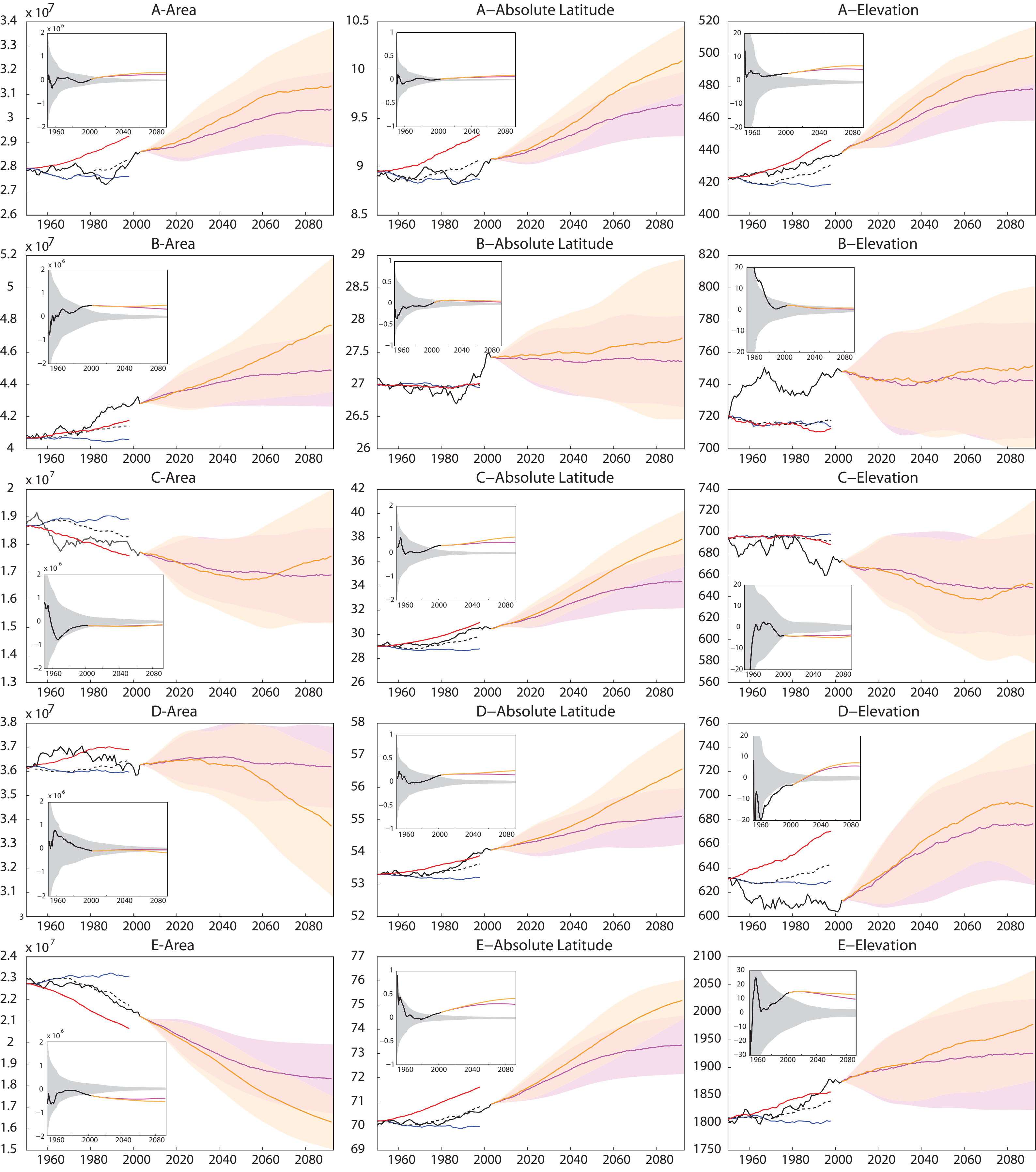
A-Area
A–Absolute Latitude
A–Elevation
B-Area
B–Absolute Latitude
B–Elevation
C-Area
C–Absolute Latitude
C–Elevation
D-Area
D–Absolute Latitude
D–Elevation
E-Area
E–Absolute Latitude
E–Elevation
